# Consideration of Gut Microbiome in Murine Models of Diseases

**DOI:** 10.3390/microorganisms9051062

**Published:** 2021-05-14

**Authors:** Chunye Zhang, Craig L. Franklin, Aaron C. Ericsson

**Affiliations:** 1 Department of Veterinary Pathobiology, University of Missouri, Columbia, MO 65201, USA; czvw9@mail.missouri.edu; 2 Mutant Mouse Resource and Research Center, University of Missouri, Columbia, MO 65201, USA; 3 Metagenomics Center, University of Missouri, Columbia, MO 65201, USA

**Keywords:** gut microbiome, virome, mouse model of disease, modulation, reproducibility, translatability

## Abstract

The gut microbiome (GM), a complex community of bacteria, viruses, protozoa, and fungi located in the gut of humans and animals, plays significant roles in host health and disease. Animal models are widely used to investigate human diseases in biomedical research and the GM within animal models can change due to the impact of many factors, such as the vendor, husbandry, and environment. Notably, variations in GM can contribute to differences in disease model phenotypes, which can result in poor reproducibility in biomedical research. Variation in the gut microbiome can also impact the translatability of animal models. For example, standard lab mice have different pathogen exposure experiences when compared to wild or pet store mice. As humans have antigen experiences that are more similar to the latter, the use of lab mice with more simplified microbiomes may not yield optimally translatable data. Additionally, the literature describes many methods to manipulate the GM and differences between these methods can also result in differing interpretations of outcomes measures. In this review, we focus on the GM as a potential contributor to the poor reproducibility and translatability of mouse models of disease. First, we summarize the important role of GM in host disease and health through different gut–organ axes and the close association between GM and disease susceptibility through colonization resistance, immune response, and metabolic pathways. Then, we focus on the variation in the microbiome in mouse models of disease and address how this variation can potentially impact disease phenotypes and subsequently influence research reproducibility and translatability. We also discuss the variations between genetic substrains as potential factors that cause poor reproducibility via their effects on the microbiome. In addition, we discuss the utility of complex microbiomes in prospective studies and how manipulation of the GM through differing transfer methods can impact model phenotypes. Lastly, we emphasize the need to explore appropriate methods of GM characterization and manipulation.

## 1. Role of Gut Microbiome in Disease

### 1.1. Gut Microbiome in Health and Disease

The term gut microbiome (GM) refers to the community of all of the microorganisms, including bacteria, viruses (virome), protozoa (protozoome), fungi (mycobiome), and their collective genetic material, that colonize and exist in the guts of all animals [1,2]. In the host, the GM plays a critical role in providing nutrition through the metabolism of dietary components [3] and the absorption of minerals [4], the maintenance of the normal function of the gut barrier [5,6], protection against pathogen infection through colonization resistance [7] and contributing to immune system development [8], drug metabolism [9] and hormone secretion [10], all of which influence the health of the host.

**Figure 1:**
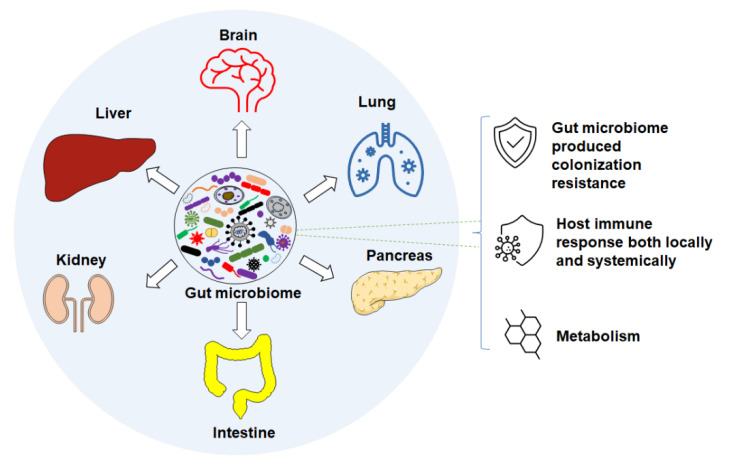
Gut microbiome associates with disease through different axes via different mechanisms such as: colonization resistance, host immune response, and metabolism.

Accumulating studies suggest that changes or differences in the GM are associated with numerous intestinal diseases, such as inflammatory bowel disease (IBD) [11,12,13], irritable bowel syndrome (IBS) [14,15,16], colon cancer [17,18], and *Clostridium difficile* infection [19,20]. Differences in GM are also associated with non-intestinal conditions via different axes (Figure 1) in human and mouse diseases. For instance, features of the GM are associated with neurodegenerative and neuropsychiatric disorders [21], such as Parkinson’s [22,23] and Alzheimer’s disease [24] through the gut–brain axis [25], respiratory diseases via the gut–lung axis [26,27], liver diseases through the gut–liver axis [28,29,30,31], cardiovascular diseases [32,33], autoimmune disorders [34,35,36,37], and others [38]. In the following subsections, we review the current knowledge of the GM of laboratory mice, and its influence on host health and disease susceptibility through colonization resistance, immune responses, and metabolic pathways.

### 1.2. Gut Microbiome and Colonization Resistance

The GM, harboring symbiotes and commensals during health, can also serve as a reservoir and transporter of pathogenic bacteria and viruses [39,40]. Pathobiont bacteria such as E. coli replicate and proliferate mainly in the gut after infection [41]. The commensal microbiome plays an essential role in protecting the host from the overgrowth of pathobionts, and the invasion of foreign pathogenic bacterial and viral infection, using different strategies collectively referred to as colonization resistance (CR). Mechanisms of CR include out-competing pathogenic bacteria for space and nutrition, and producing bactericidal factors like antimicrobial peptides [42]. Notably, conventional mice harboring a specific pathogen-free (SPF) microbiome are less susceptible to bacterial infection compared to germ-free mice [43]. Similarly, germ-free mice inoculated with Oligo-Mouse-Microbiota (OMM12), a commensal bacterial community containing 12 bacterial species originally isolated from mice, are less susceptible to infection compared to Altered Schaedler Flora (ASF)-colonized mice due to the increased colonization resistance in OMM12 mice [44]. Both examples demonstrate the protective function of colonization resistance conferred by the commensal microbiome.

### 1.3. Gut Microbiome and Immunity

Commensal bacteria protect the host not only by directly competing with pathogenic bacteria for available space and energy sources, but also indirectly through their role in immune system development. Many studies [45,46] have demonstrated the complex interaction between the gut microbiome and host immunity, including both local and systemic immune responses, in a variety of diseases.

Certain commensal bacterial species, such as segmented filamentous bacteria (SFB) [47,48,49], have been identified as potent inducers of secretory IgA (sIgA), IL-17, and defensins. The presence of these immune mediators can enhance host resistance to bacterial pathogens. For example, immunoglobulin A, produced at the mucosal surface, plays a critical role in intestinal immunity. When infection occurs, high-affinity pathogen-specific secretory IgA (sIgA) is secreted into the intestinal lumen to serve a protective function via mechanisms such as viral or bacterial toxin neutralization [50,51,52].

The presence of SFB in the gut microbiome plays a protective role against *Citrobacter rodentium* [53] and decreases host susceptibility to *Salmonella* colonization in rats [54] and *E. coli* O103 infection in rabbits [55]. sIgA plays an important role in pathogen clearance through effector functions, such as limiting pathogen growth in the gut lumen, preventing the interaction between pathogen and host intestinal mucosa, and decreasing bacteria-induced inflammatory responses in the case of *Salmonella Typhimurium* diarrhea [56] and *Shigella flexneri* infection [57].

SFB also induces the increased production of IL-17, IL-22, and the antimicrobial peptide RegIIIγ [58,59,60]. This protective T helper 17 (Th17) response has been shown to be important in the defense against *Citrobacter rodentium* infection [61]. Similarly, IL-17 and IL-22 play protective roles in *Salmonella* infection [62,63], and IL-22 can enhance the secretion of antimicrobial peptides in intestinal epithelial cells.

Another example of the association between the gut microbiome and immunity can be seen in members of the bacterial genus *Helicobacter*. *Helicobacters* serve as provocateurs to induce a potent T helper type 1 (Th1) immune response to normally commensal bacteria. In turn, *Helicobacters*, such as *H. hepaticus* and *H. bilis*, are used as disease triggers in many mouse models of gastrointestinal disease, including models of inflammatory bowel disease (IBD) and colitis-associated colorectal cancer (CAC) [64,65].

### 1.4. Gut Microbiome and Metabolites

Microbiome metabolites, such as bile acids and short-chain fatty acids (SCFAs), can maintain host health by providing nutrition and energy and modulating host immunity. These metabolites include small compounds produced directly by commensal bacteria and the end products of dietary substrates metabolized by commensal bacteria, both playing an important role in maintaining host health [66]. Primary bile acids such as chenodeoxycholic acids [67] have been demonstrated to have bactericidal activity against pathogenic bacteria, as they are associated with the increased production of host antimicrobial peptides. GM-derived secondary bile acids and symbiotic products such as propionate also hinder the colonization of bacterial pathobionts, such as *C. difficile* [68,69]. GM-derived SCFAs have also been shown to influence diseases such as obesity, Parkinson’s disease, and those that disrupt intestinal epithelial integrity [70,71,72,73]. SCFAs also have beneficial effects in terms of maintaining intestinal homeostasis through immune modulation [74]. Kang et al. [75] reported the reduced abundance of butyrate-producing bacterial species within the family *Ruminococcaceae* in Crohn’s disease (CD) patients compared to healthy individuals. These studies support the notion that the GM influences disease susceptibility through metabolites present in the lumen of the gut.

Collectively, through various direct and indirect mechanisms, such as colonization resistance, immune modulation, and modulation of host metabolism, the gut microbiome plays an essential role in both human and animal health and disease.

## 2. Factors Contributing to Gut Microbiome Variation

Considering the important role that the GM plays in host health and disease, differences in the GM between mice could result in a different disease phenotype in a given model, causing poor research reproducibility [76]. Mouse models are useful and valuable tools to investigate many disease mechanisms of, and therapeutics for, human disease. However, there are many factors that can influence the gut microbiome of mouse models.

**Figure 2:**
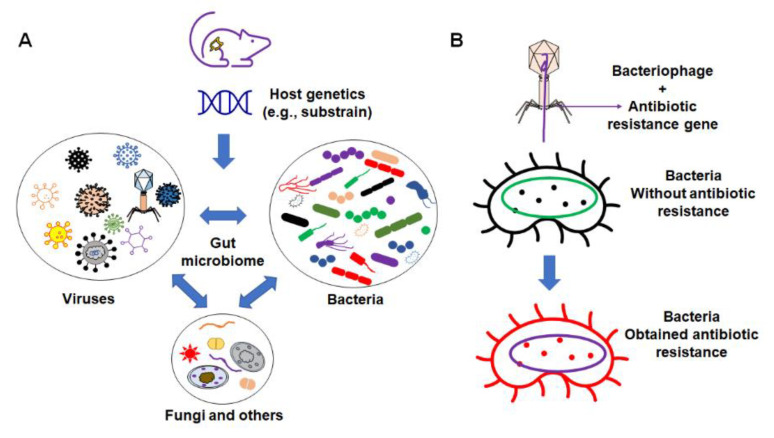
Organic factors that contribute to the variation in microbiome. (**A**) The interaction of the gut microbiome with bacteria, virus, fungi, and other components; (**B**) example of bacteriophages’ influence on commensal bacteria by transferring the antibiotic resistance gene to non-resistant bacteria.

**Figure 3:**
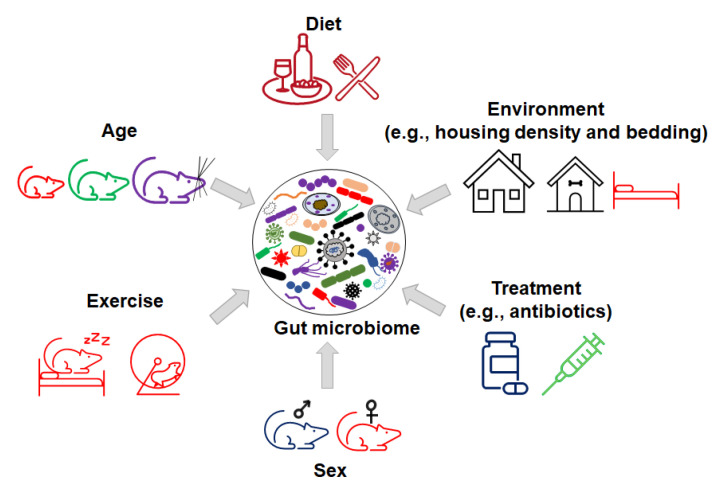
Inorganic factors influence the variation in gut microbiome.

**Figure 4:**
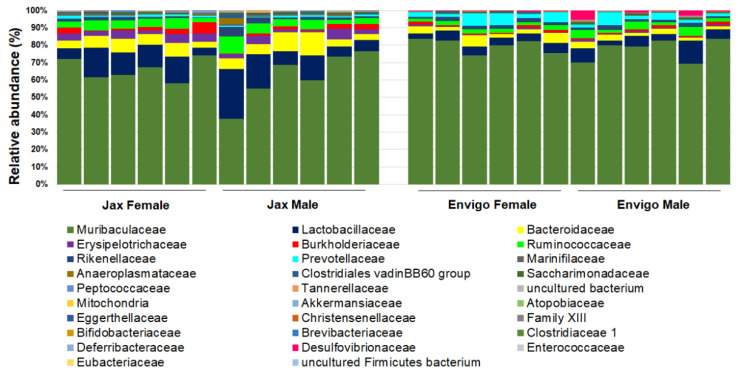
Representative GM of female (*n* = 6/vendor) and male (*n* = 6/vendor) specific pathogen-free laboratory mice from Jackson (Jax, left) or Envigo (right), annotated to the taxonomic level of family.

These factors include the interaction between different organic components within the gut microbiome (Figure 2), as well as various environmental factors (Figure 3). Organic factors that contribute to the variation in the gut microbiome include the interaction between the virome and commensal bacteria. For example, bacteriophages can transfer antibiotic resistance genes (ARG) to commensal bacteria or pathogenic bacteria in the gut through transduction. Besides the modulation of ARG, bacteriophages can also impair the intestinal barrier and result in an increase in intestinal permeability and a change in GM abundance [77]. Similarly, environmental factors as broad as the supplier or even the housing facility can alter the GM [78]. The supplier is a major factor—the GM of SPF mice from the Jackson Laboratory (Jax) and Envigo are characterized by many of the same dominant colonizers (e.g., *Muribaculaceae*), accompanied by stark differences in the relative abundance of many other families, including *Prevotellaceae*, *Ruminococcaceae*, and *Erysipelotrichaceae* (Figure 4).

In addition, many environmental factors such as diet, bedding, caging, housing density, and the mode of birth delivery can contribute to the variance in the microbiome composition between mice [79]. For example, preclinical and clinical studies demonstrate that dietary polyphenols with probiotic properties can impact GM composition, gut permeability, metabolism, and immune responses [80,81]. The consumption of polyphenols modulates the relative abundance of *Firmicutes* to *Bacteroidetes* [82]. Increased numbers of studies show that the supplementation of polyphenols from apple juice, berries, red wine, and teas can increase the relative abundance of probiotic bacteria, such as *Lactobacillus* and *Bifidobacterium*, and decrease some pathogenic bacteria, such as *H. pylori* and *C. difficile* [83]. In addition, polyphenols impact gut microbiota metabolites to exert anti-oxidative, anti-cancer, and anti-inflammatory activities [83,84].

Host genetics have also been shown to “shape” the GM [85,86]. For example, in the IL10^−/−^ mouse model of inflammatory bowel disease, different genetic backgrounds (C3H and C57/BL6) result in variations in GM colonization [87]. In summary, the variations in GM composition in contemporary mouse colonies and the multitude of factors that can modulate the GM highlight the need to consider GM as a potential cause when differences in disease phenotypes arise.

## 3. Microbiome Variation and Reproducibility of an Animal Disease Model

In addition to the aforementioned role of SFB in mucosal immune system development and the subsequent influence on *Citrobacter rodentium* colitis, there are many examples of how certain commensal bacteria can modulate host physiology and disease. For example, members of the phylum *Firmicutes* produce butyrate, which down-regulates the expression of epithelial indoleamine 2,3-dioxygenase-1 (IDO-1), an important molecule that modulates intestinal immune responses [88].

Interactions between commensal bacteria and host immune responses also have the potential to alter disease phenotypes. For example, germfree mice colonized by commensal bacterial consortia and coated with a large amount of IgA are more susceptible to colitis when compared to mice colonized by a commensal consortium with lower levels of IgA coating [89].

Thus, the variations in GM, their products and their complex interactions with the host have great potential to modulate the disease phenotypes of animal models.

## 4. Microbiome and Translatability of Mouse Models to Human Disease

Mouse models are widely used to investigate the genetic basis of human disease due to the feasibility of genetic modifications in mice [90,91,92]. In addition, the GM of mice and humans are similar in that both are made up of roughly 90% *Firmicutes* and *Bacteroidetes* [93]. The translatability of a mouse model for studying human disease refers to its ability to accurately predict the mechanism or outcomes of a human disease or condition. Considering the variability of the GM among mouse models and its influence on disease phenotypes, it follows that the translatability of a mouse model and its cognate GM should also be considered when comparing preclinical study results from mouse models to human disease [94,95]. Since differences in the mouse GM can influence host immunity and disease susceptibility and result in discrepant research results in preclinical studies, the question arises as to which preclinical results are the most translatable, or representative, of the human disease under investigation.

Recently, attention has been focused on mice from non-laboratory sources (e.g., pet stores, feral populations, and wild mice) due to the significant differences in antigen experience compared to traditional lab mice. As a result, more antigen-exposed pet store mice develop a human adult-like immune system, while the less antigen-experienced lab mice retain an infant-like immune system [96]. In a separate study, lab mice colonized with the GM of wild mice show increased resistance to influenza virus infection, and a reduced incidence of AOM/DSS-induced colorectal cancer compared to cohorts harboring the GM profile of standard lab mice [97]. These studies highlight that differences in antigen experience have a profound impact on the immune profile and associated susceptibility to a broad range of diseases.

## 5. Genetic Drift of Substrain and Disease in Mouse Model

Susceptibility to many diseases, both infectious and immune-mediated, often has an underlying genetic basis [86,98,99,100,101]. The different inbred mouse strains BALB/c and C57BL/6 have differences in their ability to produce IgA, which results in a higher diversity of microbiota in BALB/c mice compared to C57BL/6 mice [102]. Additionally, genetic drift describes the variation between different mouse substrains within the same genetic background. The genetic variation between substrains potentially impacts the diversity of the GM and the disease susceptibility of mouse models. Genetic factors play an important role in shaping the human gut microbiome as well, consequently influencing metabolism and disease susceptibility [86]. When studies were conducted using two different substrains of C57BL/6 mice (B6N and B6J), the results of select neurological function tests were significantly different between substrains [103]. Some metabolism-related diseases differ between the different substrains of C57BL/6 mice due to the mutation of the nicotinamide nucleotide transhydrogenase (*Nnt*) gene [104,105,106,107]. These examples demonstrate that differences between mouse substrains can impact disease phenotypes. Unfortunately, many of these studies were performed prior to the recognition that GM can also influence the model phenotype and, almost invariably, they were performed without consideration for differing GM (such as the profound differences seen in B6 substrains from the Jackson Laboratory and Envigo). Moving forward, when designing or troubleshooting experiments using animal models, it will be critical to consider host genetic and microbial factors as well as the complex interactions between the two.

## 6. Methodology to Investigate the Contribution of Genetics and Microbiome

As described above, both GM and host genetics can play critical roles in host disease susceptibility and there is frequent interaction between these two factors, making controlled studies difficult. To address this issue, we applied complex microbiota-targeted rederivation (CMTR) [108] to generate genetically engineered mouse models harboring distinct microbiome profiles. Simply, embryos from mice of the chosen genetic background are transferred into surrogate CD-1 dams that harbor different complex microbiome profiles. Pups thus obtain their GM during the natural process of delivery and maternal care. In this way, the complex microbiome can be faithfully transferred to any mouse strain or model for further research purposes. Using this strategy, isogenic (genetically identical) mice harboring different GMs are created and can be used to investigate the role of GM variations in model phenotypes. This approach can also be used to transfer the same GM into mice with different genetic backgrounds in order to investigate the genetic factors that may shape the microbiome. Using the described methods, we [87] successfully identified the GM as a contributing determinant in the IL-10^-/-^ IBD model, demonstrating that the variation in GM among commercial vendors can affect the disease severity. Recent studies [109,110] using a similar approach showed that the microbiome and genetics both play a critical role in the development of colon cancer in a mouse model of familial and spontaneous colon cancer. This approach can also be applied to investigate different areas, such as identifying the commensal bacteria contributing to disease, signaling pathways, drug metabolism, and treatment efficacy.

## 7. Reasons to Modulate the Microbiome in Animal Models and Potential Application

**Figure 5:**
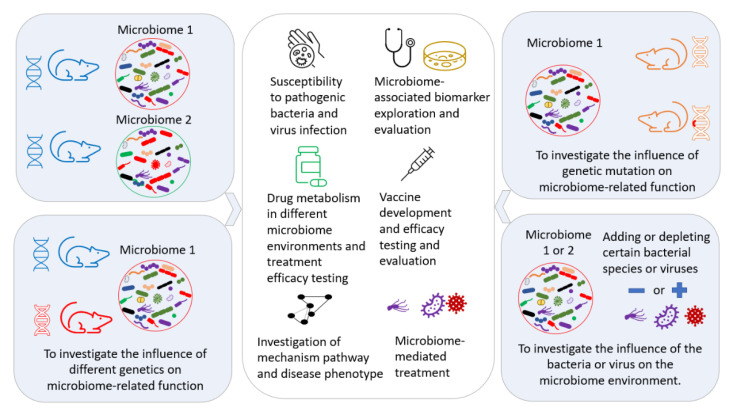
Exploration of the potential applications of microbiome transfer in biomedical research.

The manipulation and modulation of the gut microbiome are performed for different purposes, such as creating a well-controlled GM environment for further investigation of the underlining mechanisms [109], identifying the contributing component(s) of the GM [111], exploring the interactions between different commensal bacteria in the GM [112], therapeutic approaches [113,114], investigating drug metabolism for the development of precision medicine [115,116,117], and improving the reproducibility [118] of biomedical research by decreasing the variability induced by differing GMs. A controlled GM transferred to a mouse can provide a controlled GM environment in any genetically modified disease model, as illustrated in Figure 5.

### 7.1. Improve Reproducibility through a Better Understanding of Methods to Transfer the GM

**Figure 6:**
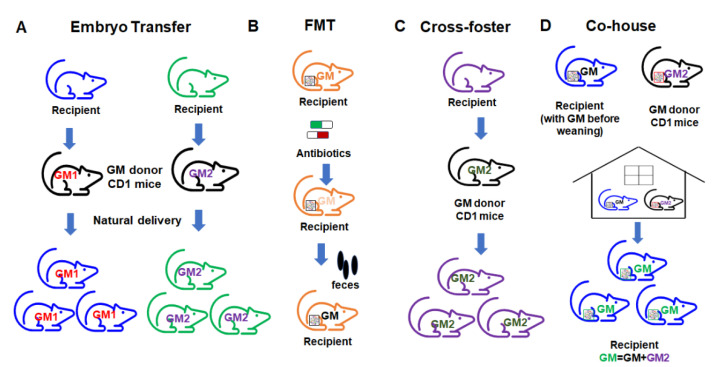
Different approaches to gut microbiome transfer. (**A**) Embryo transfer; (**B**) fecal microbiome transfer (FMT); (**C**) cross-foster method; (**D**) co-house method.

Currently, there are several different ways to transfer the gut microbiome in mouse models of disease (Figure 6). The embryo transfer (ET) method is considered the gold standard for GM transfer. For facilities where ET is not possible, researchers often use alternative methods such as fecal microbiome transfer (FMT), co-housing (CH), and cross-fostering (CF). These methods each carry certain limitations and the method of GM transfer can itself affect model outcomes. Researchers should therefore be aware of these method-based influences, control for them accordingly, and interpret the resulting data in the context of the transfer methods used. The pros and cons of each method should be considered.

In the ET approach, the embryos of the intended GM recipient mice are collected and surgically transferred to a pseudopregnant GM donor dam. The transferred embryos go through the fetal development stage in the donor GM environment. The recipient pups can then obtain the vaginal microbiome from the donor dam through natural delivery. After birth, the pups acquire the donor GM through maternal care. In this way, the complete donor GM can be transferred to the recipient mice and pups harboring the transferred GM can be used for the study. ET results in a very high-efficiency transfer of the donor GM due to exposure to the donor GM environment during the delivery process, and with maternal care. However, this method requires considerable expertise and well-trained personnel, and is relatively expensive, making it inaccessible for many labs.

FMT is a commonly used method wherein fecal or cecal contents (either frozen or freshly prepared slurries) from donors are transferred to recipient mice through gastric gavage. The advantage of FMT in animal models is the flexibility of using stored fecal or ceca contents, as well as the ease of use according to the procedure. Many mouse FMT studies use germfree mice. However, for some studies that do not use germfree mice, prior to transfer, the administration of antibiotics is often required to first deplete the microbiome in recipient mice. An additional consideration with FMT is that transfer efficacy is highly dependent on the GM richness of the donor and recipient [119].

CH is another commonly used method in the literature, wherein recipient mice are co-housed with donor mice after weaning [120,121], resulting in the transfer of the donor GM through coprophagy and grooming [119,121]. The advantage of co-housing is the ease of use and low cost. However, co-housing results in the transfer of GM after a critical pre-weaning period during which immune system development occurs and the microbiome is changing rapidly, which results in an incomplete transfer and does not capture any GM-mediated influences on the developmental processes, and associated phenotypes that dependent on some developmental processes. Additionally, the transfer efficiency of CH is low due to the fact that recipient mice already have an established GM, resulting in a hybridized GM.

CF, as a method of GM transfer, represents a third option. The recipient pups are placed with the GM donor dam within 24 h after birth, allowing the recipients to pick up most of their GM from the GM donor dam from an early age during the maternal care process. Theoretically, CF will transfer the GM with higher transfer efficiency compared to CH. CF has the advantages of ease of use and low cost compared to the ET method. However, there are some drawbacks of using the CF method, such as the requirement for timed mating, incomplete transfer due to the lack of vaginal GM transfer and the creation of a potentially hybridized GM.

### 7.2. Investigation of the Disease Mechanism and Diagnostic Biomarker

The composition of the gut microbiome has been proposed as a potential diagnostic biomarker for many diseases. The exploration of microbiome-based biomarkers has included multiple associations between the ratio between the two dominant phyla (*Firmicutes/Bacteroidetes*) as a biomarker for obesity [122] and related conditions [123,124]. Microbiome-based biomarkers have also been used to predict disease progression. For example, *Lactobacillales* and *Verrucomicrobiales* are enriched in early-stage liver fibrosis, while *Enterobacteriales* are enriched at a later stage [125]. Other examples include *Fusobacterium nucleatum* as a potential biomarker for colorectal cancer [126,127] and increases in *Bacillus* as a biomarker of lung cancer [128]. However, the variation in GM at the population level impacts the accuracy of diagnosis as a disease biomarker; thus, much work is required to fully appreciate the mechanisms underlying microbial biomarkers.

### 7.3. The Efficiency of Microbiome-Mediated Therapeutic Exploration

The exploration of gut microbiome-mediated treatment for various diseases has drawn extensive attention in the field. Beneficial microbiome components have been administered through dietary intervention, probiotic supplementation, and FMT to enhance the stability of the ecosystem or modulate the host immune response. One example is FMT as an effective microbiome-based therapeutic option [129,130,131,132] for *C. difficile* colitis. This involves the transfer of fecal material from a healthy donor to a patient with *C. difficile* bacterial overgrowth. Many other studies [133,134,135] have investigated the use of microbiome-based treatment for inflammatory bowel disease, including FMT to transfer beneficial commensal bacteria such as *Bifidobacterium* sp. One recent study [136] demonstrated the modulatory function of the microbiota in regulatory T (Treg) cell MyD88/RORγt signaling in the treatment of food allergies. Other therapeutic applications include the treatment of chronic kidney disease [137,138], autism [113,139], Parkinson’s disease [140], diabetes [141,142], obesity [143,144] and cancer [145,146,147,148,149].

Therefore, the exploration of microbiome-based therapeutic approaches is dependent on the GM environment in which they are studied. Animal models are critical to the development of new microbiome-based therapies and have the advantage of being carried out in a controlled GM (and genetic background and environment), which cannot be accomplished in human studies. A well-defined and well-controlled GM environment coupled with a well-validated microbiome modulation method is essential for the investigation of treatment approaches, and the efficacy of therapy.

The gut microbiome is associated with the immunotherapy response to treatments for cancers such as hepatocellular carcinoma [150], gastrointestinal cancer [151], lung cancer [152], and others [153]. The abundance of certain commensal bacteria, such as *Bifidobacterium longum* [154], *Akkermansia muciniphila* [152], and members of the *Ruminococcaeae* family [155], showed a significant association with the treatment efficacy. Manipulation of the microbiome, such as oral supplementation with the commensal bacteria, *Akkermansia muciniphila*, enhanced the response to immune checkpoint inhibitor treatment in a mouse model of melanoma [152].

The gut microbiome is also involved in drug metabolism through both direct and indirect processes [9,117,156,157]. Commensal bacteria transform xenobiotics (i.e., drugs) in the lumen, using enzymes to steal carbon as an energy source, and the possible effects on the parent compound include the activation of an inactive prodrug, such as irinotecan or CPT-11, via gut microbial β-glucuronidase to allow its therapeutic function, the inactivation of an active drug, such as 5-aminosalicylic acid, by gut microbial N-acetyltransferases to render it ineffective or even toxic, and an increase or reduction in the compound half-life [158,159].

Thus, the development and characterization of a novel drug and the metabolic pathway in the model host will benefit from a well-controlled microbiome environment due to the important contribution of the microbiome in drug metabolism.

## 8. Conclusions and Perspectives

Despite their limitations, mouse models are still a valuable, practical, and irreplaceable tool for studying human disease. No animal models are 100% ideal for modeling human disease. However, a better understanding of each model system can provide an improved study design and overcome the limitations associated with animal models.

Most importantly, it is necessary to consider experimental methods and platforms as factors affecting experimental reproducibility, alongside exploring novel tools to identify and investigate the different factors that influence the outcomes of each model. The application of a well-controlled GM and an appropriate transfer method to transfer the GM between genetically generated mouse models can provide the advantage of placing both the genetic background and GM under well-controlled conditions. With rigorous experimental designs, considering the GM and the methods used to manipulate the GM as experimental variables, animal models can be used more effectively to provide information that is translatable to humans in areas such as drug development and diagnostic biomarkers.

## Data Availability

All of the data were included in this manuscript.
